# Bis(2,6-diisopropyl­phen­yl) sulfite

**DOI:** 10.1107/S160053681101885X

**Published:** 2011-05-25

**Authors:** Jing-Yu Zhang, Xuehui Hou, Zheguang Wang

**Affiliations:** a School of Pharmacy, Henan University of Traditional Chinese Medicine, Zhengzhou 450008, People’s Republic of China; b Department of Quality Detection and Management, Zhengzhou College of Animal Husbandry Engineering, Zhengzhou 450011, People’s Republic of China; c College of Information and Management Science, Henan Agricultural University, Zhengzhou 450002, People’s Republic of China

## Abstract

In the title compound, C_24_H_34_O_3_S, the dihedral angle between the benzene rings is 84.62 (8)°. In the crystal, inter­molecular C—H⋯O hydrogen bonds link mol­ecules into zigzag chains running parallel to the *c* axis. The C atoms of two isopropyl groups are disordered over two sets of sites with occupancy ratios of 0.858 (9):0.142 (9) and 0.61 (5):0.39 (5).

## Related literature

For applications of propofol (2,6-diisopropyl­phenol) and its derivatives in the biochemical and pharmaceutical fields, see: Zhang *et al.* (1999 ▶); Lubarsky *et al.* (2007 ▶).
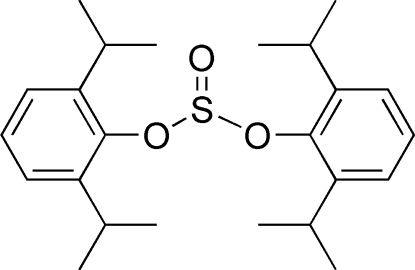


## Experimental

### 

#### Crystal data

C_24_H_34_O_3_S
*M* *_r_* = 402.57
Orthorhombic, 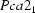

*a* = 14.2083 (15) Å
*b* = 16.3332 (17) Å
*c* = 10.1321 (10) Å
*V* = 2351.3 (4) Å^3^
*Z* = 4
Mo *K*α radiation
μ = 0.16 mm^−1^
*T* = 293 K
0.43 × 0.40 × 0.20 mm

#### Data collection

Bruker  SMART CCD area-detector diffractometer
Absorption correction: multi-scan (*SADABS*; Sheldrick, 1996 ▶) *T*_min_ = 0.935, *T*_max_ = 0.969
9296 measured reflections
3302 independent reflections
2428 reflections with *I* > 2σ(*I*)
*R*_int_ = 0.034

#### Refinement

*R*[*F* ^2^ > 2σ(*F* ^2^)] = 0.044
*wR*(*F* ^2^) = 0.119
*S* = 1.05
3302 reflections
267 parameters
11 restraints
H-atom parameters constrained
Δρ_max_ = 0.38 e Å^−3^
Δρ_min_ = −0.16 e Å^−3^
Absolute structure: Flack (1983) ▶, 1103 Friedel pairs
Flack parameter: −0.01 (10)

### 

Data collection: *SMART* (Siemens, 1996 ▶); cell refinement: *SAINT* (Siemens, 1996 ▶); data reduction: *SAINT*; program(s) used to solve structure: *SHELXS97* (Sheldrick, 2008 ▶); program(s) used to refine structure: *SHELXL97* (Sheldrick, 2008 ▶); molecular graphics: *SHELXTL* (Sheldrick, 2008 ▶); software used to prepare material for publication: *SHELXTL*.

## Supplementary Material

Crystal structure: contains datablocks I, global. DOI: 10.1107/S160053681101885X/rz2600sup1.cif

Structure factors: contains datablocks I. DOI: 10.1107/S160053681101885X/rz2600Isup2.hkl

Supplementary material file. DOI: 10.1107/S160053681101885X/rz2600Isup3.cml

Additional supplementary materials:  crystallographic information; 3D view; checkCIF report

## Figures and Tables

**Table 1:** Hydrogen-bond geometry (Å, °)

*D*—H⋯*A*	*D*—H	H⋯*A*	*D*⋯*A*	*D*—H⋯*A*
C5—H5⋯O1^i^	0.93	2.57	3.413 (4)	151

Symmetry code: (i) 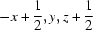.

**Fig. 1.**
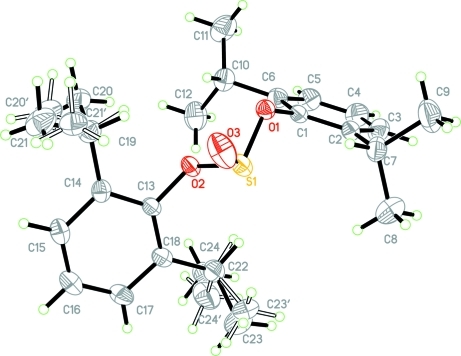
The molecular structure of the compound, with atom labels and 50% probability displacement ellipsoids.

**Fig. 2.**
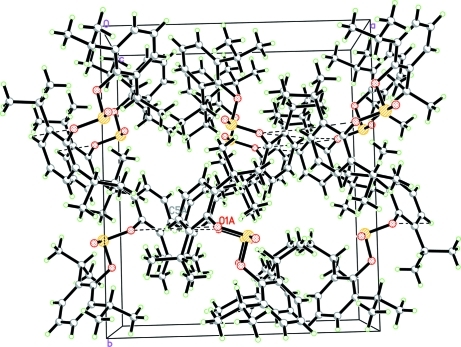
Crystal packing of the title compound showing chains formed by hydrogen bonds (dashed lines) running parallel to the c axis. Only the major components of disorder are shown.

## supplementary crystallographic information

### Comment

Propofol (2,6-diisopropylphenol) derivatives are an important class of compounds having a broad spectrum of applications in the biochemical and pharmaceutical fields (Zhang *et al.*, 1999; Lubarsky *et al.*, 2007). In order to develop new applications for propofol and its derivatives, structural modifications of propofol have been extensively investigated. As a contribution in this field, we report here the crystal structure of the title compound.

The molecular structure of title compound is shown in Fig. 1. The dihedral angle formed by the benzene rings 84.62 (8)°. In the crystal packing (Fig. 2), intermolecular C—H···O hydrogen bonds (Table 1) link molecules into zigzag chains running parallel to the *c* axis.

### Experimental

To a solution of 2,6-diisopropylphenol (178 g, 1.00 mol) in tetrahydrofuran (1.00 l), SOCl_2_ (59 g, 0.50 mol) was added. The mixture was stirred at 0°C for 5 h. Then H_2_O (20 ml) was added to the mixture, followed by extraction with toluene. The organic phase was concentrated and purified by crystallization from ethyl acetate. Colourless crystals suitable for X-ray analysis were obtained on slow evaporation of the solvent.

### Refinement

All H atoms were placed geometrically and treated as riding on their parent atoms, with C—H = 0.93–0.96 Å and *U*_iso_(H) = 1.2*U*_eq_(C) or 1.5*U*_eq_(C) for methyl H atoms. Two isopropyl groups are disordered over two sets of sites with refined occupancy ratios of 0.858 (9):0.142 (9) and 0.61 (5):0.39 (5). During the refinement, the C—C distances involving the disordered atoms were constrained to be 1.54 (1) Å.

### Crystal data

**Table:**

C_24_H_34_O_3_S	*F*(000) = 872
*M**_r_* = 402.57	*D*_x_ = 1.137 Mg m^−^^3^
Orthorhombic, *P**c**a*2_1_	Mo *K*α radiation, λ = 0.71073 Å
Hall symbol: P 2c -2ac	Cell parameters from 2762 reflections
*a* = 14.2083 (15) Å	θ = 2.8–21.7°
*b* = 16.3332 (17) Å	µ = 0.16 mm^−^^1^
*c* = 10.1321 (10) Å	*T* = 293 K
*V* = 2351.3 (4) Å^3^	Block, colourless
*Z* = 4	0.43 × 0.40 × 0.20 mm

### Data collection

**Table:**

Bruker SMART CCD area-detector diffractometer	3302 independent reflections
Radiation source: fine-focus sealed tube	2428 reflections with *I* > 2σ(*I*)
graphite	*R*_int_ = 0.034
φ and ω scans	θ_max_ = 25.0°, θ_min_ = 1.9°
Absorption correction: multi-scan (*SADABS*; Sheldrick, 1996)	*h* = −15→16
*T*_min_ = 0.935, *T*_max_ = 0.969	*k* = −19→18
9296 measured reflections	*l* = −9→12

### Refinement

**Table:**

Refinement on *F*^2^	Secondary atom site location: difference Fourier map
Least-squares matrix: full	Hydrogen site location: inferred from neighbouring sites
*R*[*F*^2^ > 2σ(*F*^2^)] = 0.044	H-atom parameters constrained
*wR*(*F*^2^) = 0.119	*w* = 1/[σ^2^(*F*_o_^2^) + (0.0616*P*)^2^ + 0.0966*P*] where *P* = (*F*_o_^2^ + 2*F*_c_^2^)/3
*S* = 1.05	(Δ/σ)_max_ < 0.001
3302 reflections	Δρ_max_ = 0.38 e Å^−^^3^
267 parameters	Δρ_min_ = −0.16 e Å^−^^3^
11 restraints	Absolute structure: Flack (1983), 1103 Friedel pairs
Primary atom site location: structure-invariant direct methods	Flack parameter: −0.01 (10)

### Special details

**Table:**

Geometry. All e.s.d.'s (except the e.s.d. in the dihedral angle between two l.s. planes) are estimated using the full covariance matrix. The cell e.s.d.'s are taken into account individually in the estimation of e.s.d.'s in distances, angles and torsion angles; correlations between e.s.d.'s in cell parameters are only used when they are defined by crystal symmetry. An approximate (isotropic) treatment of cell e.s.d.'s is used for estimating e.s.d.'s involving l.s. planes.
Refinement. Refinement of *F*^2^ against ALL reflections. The weighted *R*-factor *wR* and goodness of fit *S* are based on *F*^2^, conventional *R*-factors *R* are based on *F*, with *F* set to zero for negative *F*^2^. The threshold expression of *F*^2^ > σ(*F*^2^) is used only for calculating *R*-factors(gt) *etc*. and is not relevant to the choice of reflections for refinement. *R*-factors based on *F*^2^ are statistically about twice as large as those based on *F*, and *R*- factors based on ALL data will be even larger.

### Fractional atomic coordinates and isotropic or equivalent isotropic displacement parameters (Å^2^)

**Table:**

	*x*	*y*	*z*	*U*_iso_*/*U*_eq_	Occ. (<1)
S1	0.52350 (6)	0.68862 (5)	0.76210 (11)	0.0512 (3)	
O1	0.41636 (13)	0.65661 (11)	0.7617 (2)	0.0411 (5)	
O2	0.49612 (14)	0.77870 (12)	0.8211 (2)	0.0434 (5)	
O3	0.5471 (2)	0.69638 (16)	0.6283 (3)	0.0839 (10)	
C1	0.3791 (2)	0.62106 (18)	0.8793 (3)	0.0414 (8)	
C2	0.4071 (2)	0.54182 (19)	0.9131 (3)	0.0475 (9)	
C3	0.3640 (3)	0.5082 (2)	1.0244 (4)	0.0655 (11)	
H3	0.3809	0.4559	1.0517	0.079*	
C4	0.2976 (3)	0.5505 (3)	1.0940 (4)	0.0700 (11)	
H4	0.2696	0.5263	1.1672	0.084*	
C5	0.2718 (3)	0.6278 (2)	1.0577 (4)	0.0625 (10)	
H5	0.2269	0.6556	1.1071	0.075*	
C6	0.3116 (2)	0.6660 (2)	0.9481 (4)	0.0487 (8)	
C7	0.4794 (3)	0.4944 (2)	0.8367 (4)	0.0580 (10)	
H7	0.4967	0.5268	0.7589	0.070*	
C8	0.5690 (3)	0.4806 (3)	0.9188 (5)	0.0826 (13)	
H8A	0.5924	0.5324	0.9495	0.124*	
H8B	0.6159	0.4545	0.8651	0.124*	
H8C	0.5546	0.4464	0.9931	0.124*	
C9	0.4408 (4)	0.4119 (2)	0.7885 (5)	0.0989 (16)	
H9A	0.4237	0.3789	0.8630	0.148*	
H9B	0.4882	0.3842	0.7378	0.148*	
H9C	0.3863	0.4211	0.7344	0.148*	
C10	0.2805 (2)	0.7509 (2)	0.9078 (4)	0.0555 (9)	
H10	0.3119	0.7645	0.8244	0.067*	
C11	0.1758 (3)	0.7547 (3)	0.8849 (5)	0.0856 (14)	
H11A	0.1435	0.7452	0.9667	0.128*	
H11B	0.1580	0.7135	0.8221	0.128*	
H11C	0.1592	0.8077	0.8514	0.128*	
C12	0.3108 (3)	0.8143 (2)	1.0108 (5)	0.0834 (14)	
H12A	0.2802	0.8028	1.0932	0.125*	
H12B	0.2933	0.8681	0.9812	0.125*	
H12C	0.3778	0.8118	1.0224	0.125*	
C13	0.5700 (2)	0.83653 (17)	0.8272 (3)	0.0389 (7)	
C14	0.5699 (2)	0.89670 (17)	0.7302 (3)	0.0436 (8)	
C15	0.6400 (2)	0.95698 (18)	0.7404 (4)	0.0527 (9)	
H15	0.6434	0.9984	0.6777	0.063*	
C16	0.7037 (3)	0.9551 (2)	0.8426 (4)	0.0593 (10)	
H16	0.7496	0.9956	0.8480	0.071*	
C17	0.7008 (2)	0.8946 (2)	0.9368 (4)	0.0576 (10)	
H17	0.7450	0.8946	1.0045	0.069*	
C18	0.6329 (2)	0.83355 (19)	0.9324 (3)	0.0466 (9)	
C19	0.4951 (2)	0.8998 (2)	0.6238 (3)	0.0538 (10)	
H19	0.4796	0.8430	0.6011	0.065*	0.858 (9)
H19'	0.4526	0.8531	0.6354	0.065*	0.142 (9)
C20	0.4062 (3)	0.9389 (4)	0.6776 (6)	0.0682 (16)	0.858 (9)
H20A	0.4199	0.9933	0.7075	0.102*	0.858 (9)
H20B	0.3828	0.9069	0.7500	0.102*	0.858 (9)
H20C	0.3594	0.9411	0.6093	0.102*	0.858 (9)
C21	0.5251 (4)	0.9419 (5)	0.4970 (5)	0.087 (2)	0.858 (9)
H21A	0.5815	0.9169	0.4642	0.130*	0.858 (9)
H21B	0.5366	0.9989	0.5143	0.130*	0.858 (9)
H21C	0.4760	0.9367	0.4324	0.130*	0.858 (9)
C22	0.6297 (3)	0.7684 (2)	1.0382 (4)	0.0625 (10)	
H22	0.5803	0.7298	1.0123	0.075*	0.61 (5)
H22'	0.5704	0.7383	1.0302	0.075*	0.39 (5)
C23	0.721 (2)	0.719 (3)	1.050 (3)	0.118 (5)	0.61 (5)
H23D	0.7406	0.7015	0.9645	0.177*	0.61 (5)
H23E	0.7095	0.6718	1.1050	0.177*	0.61 (5)
H23F	0.7685	0.7523	1.0897	0.177*	0.61 (5)
C24	0.598 (2)	0.8068 (17)	1.1701 (16)	0.097 (5)	0.61 (5)
H24D	0.6454	0.8442	1.2006	0.145*	0.61 (5)
H24E	0.5891	0.7644	1.2344	0.145*	0.61 (5)
H24F	0.5399	0.8357	1.1572	0.145*	0.61 (5)
C20'	0.442 (2)	0.9801 (14)	0.647 (4)	0.0682 (16)	0.142 (9)
H20D	0.4739	1.0240	0.6029	0.102*	0.142 (9)
H20E	0.4398	0.9913	0.7403	0.102*	0.142 (9)
H20F	0.3791	0.9753	0.6137	0.102*	0.142 (9)
C21'	0.539 (3)	0.886 (3)	0.4883 (19)	0.087 (2)	0.142 (9)
H21D	0.5658	0.9361	0.4568	0.130*	0.142 (9)
H21E	0.4915	0.8675	0.4278	0.130*	0.142 (9)
H21F	0.5875	0.8450	0.4949	0.130*	0.142 (9)
C23'	0.715 (4)	0.714 (4)	1.007 (5)	0.118 (5)	0.39 (5)
H23A	0.7720	0.7440	1.0249	0.177*	0.39 (5)
H23B	0.7138	0.6983	0.9161	0.177*	0.39 (5)
H23C	0.7133	0.6660	1.0617	0.177*	0.39 (5)
C24'	0.637 (3)	0.804 (3)	1.178 (2)	0.097 (5)	0.39 (5)
H24A	0.6997	0.8243	1.1922	0.145*	0.39 (5)
H24B	0.6242	0.7613	1.2414	0.145*	0.39 (5)
H24C	0.5926	0.8473	1.1886	0.145*	0.39 (5)

### Atomic displacement parameters (Å^2^)

**Table:**

	*U*^11^	*U*^22^	*U*^33^	*U*^12^	*U*^13^	*U*^23^
S1	0.0388 (4)	0.0420 (4)	0.0729 (6)	−0.0042 (4)	0.0067 (5)	−0.0110 (5)
O1	0.0420 (12)	0.0407 (10)	0.0407 (12)	−0.0053 (8)	−0.0003 (11)	0.0035 (11)
O2	0.0373 (12)	0.0358 (11)	0.0570 (14)	−0.0053 (9)	0.0057 (11)	−0.0053 (10)
O3	0.096 (2)	0.0736 (18)	0.082 (2)	−0.0226 (16)	0.0398 (18)	−0.0178 (15)
C1	0.0405 (18)	0.0396 (17)	0.0440 (19)	−0.0102 (14)	−0.0060 (15)	0.0020 (15)
C2	0.045 (2)	0.0429 (18)	0.054 (2)	−0.0089 (15)	−0.0068 (17)	0.0074 (16)
C3	0.067 (3)	0.062 (2)	0.068 (3)	−0.003 (2)	−0.003 (2)	0.022 (2)
C4	0.062 (3)	0.083 (3)	0.064 (3)	−0.013 (2)	0.007 (2)	0.023 (2)
C5	0.054 (2)	0.074 (2)	0.060 (3)	−0.002 (2)	0.010 (2)	0.005 (2)
C6	0.042 (2)	0.053 (2)	0.051 (2)	−0.0019 (16)	0.0020 (17)	−0.0001 (16)
C7	0.072 (3)	0.0419 (19)	0.060 (2)	0.0046 (17)	−0.002 (2)	0.0019 (17)
C8	0.070 (3)	0.091 (3)	0.086 (3)	0.024 (2)	−0.005 (3)	0.006 (3)
C9	0.136 (4)	0.051 (2)	0.110 (4)	−0.010 (2)	−0.010 (4)	−0.012 (3)
C10	0.051 (2)	0.054 (2)	0.062 (2)	0.0066 (17)	0.0132 (19)	0.0029 (18)
C11	0.060 (3)	0.095 (3)	0.102 (4)	0.024 (2)	0.015 (3)	0.018 (3)
C12	0.105 (4)	0.062 (3)	0.083 (3)	0.003 (2)	0.019 (3)	−0.007 (2)
C13	0.0318 (17)	0.0358 (16)	0.0490 (19)	−0.0033 (14)	0.0008 (16)	−0.0055 (14)
C14	0.0479 (19)	0.0361 (15)	0.047 (2)	0.0045 (15)	0.0081 (15)	−0.0023 (14)
C15	0.061 (2)	0.0413 (17)	0.056 (2)	−0.0073 (16)	0.015 (2)	0.0003 (16)
C16	0.053 (2)	0.054 (2)	0.070 (3)	−0.0172 (17)	0.008 (2)	−0.009 (2)
C17	0.054 (2)	0.066 (2)	0.053 (2)	−0.0174 (19)	−0.0058 (19)	−0.0086 (19)
C18	0.041 (2)	0.0476 (19)	0.052 (2)	−0.0040 (15)	−0.0001 (17)	−0.0054 (15)
C19	0.055 (2)	0.052 (2)	0.055 (2)	0.0037 (17)	−0.0037 (18)	0.0001 (17)
C20	0.046 (3)	0.075 (4)	0.084 (3)	0.006 (3)	−0.002 (3)	0.011 (3)
C21	0.070 (3)	0.131 (6)	0.059 (3)	0.007 (4)	0.002 (3)	0.031 (4)
C22	0.070 (3)	0.060 (2)	0.057 (2)	−0.013 (2)	−0.013 (2)	0.0120 (19)
C23	0.077 (5)	0.122 (6)	0.154 (18)	0.010 (5)	−0.029 (9)	0.074 (13)
C24	0.144 (17)	0.091 (4)	0.056 (3)	−0.045 (12)	−0.022 (6)	0.007 (3)
C20'	0.046 (3)	0.075 (4)	0.084 (3)	0.006 (3)	−0.002 (3)	0.011 (3)
C21'	0.070 (3)	0.131 (6)	0.059 (3)	0.007 (4)	0.002 (3)	0.031 (4)
C23'	0.077 (5)	0.122 (6)	0.154 (18)	0.010 (5)	−0.029 (9)	0.074 (13)
C24'	0.144 (17)	0.091 (4)	0.056 (3)	−0.045 (12)	−0.022 (6)	0.007 (3)

### Geometric parameters (Å, °)

**Table:**

S1—O3	1.403 (3)	C16—H16	0.9300
S1—O1	1.610 (2)	C17—C18	1.389 (4)
S1—O2	1.635 (2)	C17—H17	0.9300
O1—C1	1.427 (4)	C18—C22	1.510 (5)
O2—C13	1.413 (4)	C19—C21	1.518 (5)
C1—C6	1.395 (5)	C19—C20	1.518 (5)
C1—C2	1.397 (4)	C19—C21'	1.525 (10)
C2—C3	1.396 (5)	C19—C20'	1.533 (10)
C2—C7	1.501 (5)	C19—H19	0.9800
C3—C4	1.365 (6)	C19—H19'	0.9800
C3—H3	0.9300	C20—H20A	0.9600
C4—C5	1.367 (5)	C20—H20B	0.9600
C4—H4	0.9300	C20—H20C	0.9600
C5—C6	1.393 (5)	C21—H21A	0.9600
C5—H5	0.9300	C21—H21B	0.9600
C6—C10	1.512 (5)	C21—H21C	0.9600
C7—C9	1.534 (5)	C22—C23	1.528 (8)
C7—C8	1.537 (5)	C22—C24'	1.535 (9)
C7—H7	0.9800	C22—C23'	1.537 (9)
C8—H8A	0.9600	C22—C24	1.543 (8)
C8—H8B	0.9600	C22—H22	0.9800
C8—H8C	0.9600	C22—H22'	0.9800
C9—H9A	0.9600	C23—H23D	0.9600
C9—H9B	0.9600	C23—H23E	0.9600
C9—H9C	0.9600	C23—H23F	0.9600
C10—C11	1.506 (5)	C24—H24D	0.9600
C10—C12	1.532 (6)	C24—H24E	0.9600
C10—H10	0.9800	C24—H24F	0.9600
C11—H11A	0.9600	C20'—H20D	0.9600
C11—H11B	0.9600	C20'—H20E	0.9600
C11—H11C	0.9600	C20'—H20F	0.9600
C12—H12A	0.9600	C21'—H21D	0.9600
C12—H12B	0.9600	C21'—H21E	0.9600
C12—H12C	0.9600	C21'—H21F	0.9600
C13—C14	1.390 (4)	C23'—H23A	0.9600
C13—C18	1.392 (5)	C23'—H23B	0.9600
C14—C15	1.405 (5)	C23'—H23C	0.9600
C14—C19	1.514 (5)	C24'—H24A	0.9600
C15—C16	1.375 (5)	C24'—H24B	0.9600
C15—H15	0.9300	C24'—H24C	0.9600
C16—C17	1.375 (5)		
			
O3—S1—O1	104.66 (17)	C20—C19—C21'	136.6 (15)
O3—S1—O2	109.22 (15)	C14—C19—C20'	105.2 (13)
O1—S1—O2	93.91 (10)	C21—C19—C20'	83.2 (15)
C1—O1—S1	118.74 (18)	C21'—C19—C20'	118.0 (19)
C13—O2—S1	116.15 (18)	C14—C19—H19	107.1
C6—C1—C2	124.2 (3)	C21—C19—H19	107.1
C6—C1—O1	117.3 (3)	C20—C19—H19	107.1
C2—C1—O1	118.4 (3)	C21'—C19—H19	74.9
C3—C2—C1	115.9 (3)	C20'—C19—H19	137.3
C3—C2—C7	121.0 (3)	C14—C19—H19'	108.7
C1—C2—C7	123.2 (3)	C21—C19—H19'	128.9
C4—C3—C2	121.5 (4)	C20—C19—H19'	76.7
C4—C3—H3	119.2	C21'—C19—H19'	104.2
C2—C3—H3	119.2	C20'—C19—H19'	110.1
C3—C4—C5	120.9 (4)	C19—C20—H20A	109.5
C3—C4—H4	119.6	C19—C20—H20B	109.5
C5—C4—H4	119.6	H20A—C20—H20B	109.5
C4—C5—C6	121.4 (4)	C19—C20—H20C	109.5
C4—C5—H5	119.3	H20A—C20—H20C	109.5
C6—C5—H5	119.3	H20B—C20—H20C	109.5
C5—C6—C1	116.2 (3)	C19—C21—H21A	109.5
C5—C6—C10	120.5 (3)	C19—C21—H21B	109.5
C1—C6—C10	123.3 (3)	H21A—C21—H21B	109.5
C2—C7—C9	111.9 (3)	C19—C21—H21C	109.5
C2—C7—C8	111.3 (3)	H21A—C21—H21C	109.5
C9—C7—C8	109.9 (3)	H21B—C21—H21C	109.5
C2—C7—H7	107.9	C18—C22—C23	113.9 (18)
C9—C7—H7	107.9	C18—C22—C24'	112.9 (19)
C8—C7—H7	107.9	C23—C22—C24'	93.6 (18)
C7—C8—H8A	109.5	C18—C22—C23'	104 (3)
C7—C8—H8B	109.5	C24'—C22—C23'	110.4 (13)
H8A—C8—H8B	109.5	C18—C22—C24	109.7 (11)
C7—C8—H8C	109.5	C23—C22—C24	113.0 (8)
H8A—C8—H8C	109.5	C23'—C22—C24	129.9 (18)
H8B—C8—H8C	109.5	C18—C22—H22	106.6
C7—C9—H9A	109.5	C23—C22—H22	106.6
C7—C9—H9B	109.5	C24'—C22—H22	122.7
H9A—C9—H9B	109.5	C23'—C22—H22	98.0
C7—C9—H9C	109.5	C24—C22—H22	106.6
H9A—C9—H9C	109.5	C18—C22—H22'	108.7
H9B—C9—H9C	109.5	C23—C22—H22'	117.8
C11—C10—C6	111.5 (3)	C24'—C22—H22'	109.0
C11—C10—C12	110.8 (3)	C23'—C22—H22'	111.9
C6—C10—C12	110.8 (3)	C24—C22—H22'	91.5
C11—C10—H10	107.9	C22—C23—H23D	109.5
C6—C10—H10	107.9	C22—C23—H23E	109.5
C12—C10—H10	107.9	H23D—C23—H23E	109.5
C10—C11—H11A	109.5	C22—C23—H23F	109.5
C10—C11—H11B	109.5	H23D—C23—H23F	109.5
H11A—C11—H11B	109.5	H23E—C23—H23F	109.5
C10—C11—H11C	109.5	C22—C24—H24D	109.5
H11A—C11—H11C	109.5	C22—C24—H24E	109.5
H11B—C11—H11C	109.5	H24D—C24—H24E	109.5
C10—C12—H12A	109.5	C22—C24—H24F	109.5
C10—C12—H12B	109.5	H24D—C24—H24F	109.5
H12A—C12—H12B	109.5	H24E—C24—H24F	109.5
C10—C12—H12C	109.5	C19—C20'—H20D	109.5
H12A—C12—H12C	109.5	C19—C20'—H20E	109.5
H12B—C12—H12C	109.5	H20D—C20'—H20E	109.5
C14—C13—C18	124.5 (3)	C19—C20'—H20F	109.5
C14—C13—O2	116.2 (3)	H20D—C20'—H20F	109.5
C18—C13—O2	119.1 (3)	H20E—C20'—H20F	109.5
C13—C14—C15	116.3 (3)	C19—C21'—H21D	109.5
C13—C14—C19	121.8 (3)	C19—C21'—H21E	109.5
C15—C14—C19	121.8 (3)	H21D—C21'—H21E	109.5
C16—C15—C14	120.5 (3)	C19—C21'—H21F	109.5
C16—C15—H15	119.8	H21D—C21'—H21F	109.5
C14—C15—H15	119.8	H21E—C21'—H21F	109.5
C15—C16—C17	121.2 (3)	C22—C23'—H23A	109.5
C15—C16—H16	119.4	C22—C23'—H23B	109.5
C17—C16—H16	119.4	H23A—C23'—H23B	109.5
C16—C17—C18	121.0 (3)	C22—C23'—H23C	109.5
C16—C17—H17	119.5	H23A—C23'—H23C	109.5
C18—C17—H17	119.5	H23B—C23'—H23C	109.5
C17—C18—C13	116.5 (3)	C22—C24'—H24A	109.5
C17—C18—C22	120.2 (3)	C22—C24'—H24B	109.5
C13—C18—C22	123.3 (3)	H24A—C24'—H24B	109.5
C14—C19—C21	114.9 (3)	C22—C24'—H24C	109.5
C14—C19—C20	110.0 (3)	H24A—C24'—H24C	109.5
C21—C19—C20	110.3 (4)	H24B—C24'—H24C	109.5
C14—C19—C21'	110.4 (15)		
			
O3—S1—O1—C1	162.9 (2)	C18—C13—C14—C15	1.2 (5)
O2—S1—O1—C1	−86.0 (2)	O2—C13—C14—C15	176.5 (3)
O3—S1—O2—C13	−66.3 (3)	C18—C13—C14—C19	−175.6 (3)
O1—S1—O2—C13	−173.3 (2)	O2—C13—C14—C19	−0.3 (4)
S1—O1—C1—C6	109.4 (3)	C13—C14—C15—C16	−0.5 (5)
S1—O1—C1—C2	−75.1 (3)	C19—C14—C15—C16	176.3 (3)
C6—C1—C2—C3	−0.9 (5)	C14—C15—C16—C17	0.2 (5)
O1—C1—C2—C3	−176.1 (3)	C15—C16—C17—C18	−0.5 (6)
C6—C1—C2—C7	179.4 (3)	C16—C17—C18—C13	1.1 (5)
O1—C1—C2—C7	4.3 (4)	C16—C17—C18—C22	−178.9 (3)
C1—C2—C3—C4	0.9 (6)	C14—C13—C18—C17	−1.5 (5)
C7—C2—C3—C4	−179.5 (4)	O2—C13—C18—C17	−176.6 (3)
C2—C3—C4—C5	−0.7 (6)	C14—C13—C18—C22	178.5 (3)
C3—C4—C5—C6	0.5 (6)	O2—C13—C18—C22	3.3 (5)
C4—C5—C6—C1	−0.5 (5)	C13—C14—C19—C21	−154.7 (4)
C4—C5—C6—C10	178.7 (3)	C15—C14—C19—C21	28.7 (6)
C2—C1—C6—C5	0.8 (5)	C13—C14—C19—C20	80.2 (4)
O1—C1—C6—C5	176.0 (3)	C15—C14—C19—C20	−96.4 (4)
C2—C1—C6—C10	−178.4 (3)	C13—C14—C19—C21'	−115.8 (19)
O1—C1—C6—C10	−3.2 (5)	C15—C14—C19—C21'	67.6 (19)
C3—C2—C7—C9	56.8 (5)	C13—C14—C19—C20'	115.8 (16)
C1—C2—C7—C9	−123.6 (4)	C15—C14—C19—C20'	−60.8 (16)
C3—C2—C7—C8	−66.6 (5)	C17—C18—C22—C23	−58.4 (16)
C1—C2—C7—C8	113.1 (4)	C13—C18—C22—C23	121.6 (16)
C5—C6—C10—C11	−56.2 (5)	C17—C18—C22—C24'	47 (2)
C1—C6—C10—C11	122.9 (4)	C13—C18—C22—C24'	−133.1 (19)
C5—C6—C10—C12	67.6 (4)	C17—C18—C22—C23'	−73 (3)
C1—C6—C10—C12	−113.2 (4)	C13—C18—C22—C23'	107 (3)
S1—O2—C13—C14	103.1 (3)	C17—C18—C22—C24	69.4 (15)
S1—O2—C13—C18	−81.3 (3)	C13—C18—C22—C24	−110.6 (15)

### Hydrogen-bond geometry (Å, °)

**Table:**

*D*—H···*A*	*D*—H	H···*A*	*D*···*A*	*D*—H···*A*
C5—H5···O1^i^	0.93	2.57	3.413 (4)	151

Symmetry codes: (i) −*x*+1/2, *y*, *z*+1/2.

## References

[bb6] Flack, H. D. (1983). *Acta Cryst.* A**39**, 876–881.

[bb1] Lubarsky, D. A., Candiotti, K. & Harris, E. (2007). *J. Clin. Anesthesia*, **19**, 397–404. 10.1016/j.jclinane.2006.11.006 17869995

[bb2] Sheldrick, G. M. (1996). *SADABS* University of Göttingen, Germany.

[bb3] Sheldrick, G. M. (2008). *Acta Cryst.* A**64**, 112–122. 10.1107/S0108767307043930 18156677

[bb4] Siemens (1996). *SMART* and *SAINT* Siemens Analytical X-ray Instruments Inc., Madison, Wisconsin, USA.

[bb5] Zhang, S., Hu, X. & Liu, Y. (1999). *Herald Med.* **18**, 354–359.

